# Round the Hospitals

**Published:** 1921-07-23

**Authors:** 


### ROUND THE HOSPITALS.

It is good news that the rules of the General Nursing Council for registering existing and intermediate nurses have been signed by the Minister of Health. They have now to be presented to both Houses of Parliament and to lie on the table for twenty-one days. It is therefore safe to assume that within the next month or so the Register will be open. Nurses should watch for this announcement in the daily and nursing papers, which will instruct them how to make application. For the moment, and to facilitate the immediate opening of the English Register, Rule 16, which provides for reciprocal training with Ireland, subject to a uniform standard of qualification, has been omitted. Negotiations are in progress between the Minister of Health and Scotland, and we understand a settlement is in sight. We note that a good deal of agitation is going on in Scotland with a view to

**ROUND THE HOSPITALS**—(*continued*).

sustaining the claim of Scottish fever nurses to a place on the General Register. The Glasgow Town Council considers that the placing of the fever nurses in a Supplementary Register, in place of the General Register, is "derogatory."

Training-schools will shortly be in possession of the amended Syllabus of training, and it is hoped that the adjustments which have been made will do much to accommodate it to the requirements and capabilities of the various schools. An important point to bear in mind is that the Syllabus of training is not the examination syllabus. The latter is not yet ready to issue, but it is not desirable to keep back the training Syllabus as amended, in view of the fact that if the first State examination is to be held in July 1924 training must begin at once. An important suggestion which the Syllabus of training will contain is that until the General Nursing Council arranges for a preliminary examination (which it has in contemplation, we understand, at as early a date as circumstances permit) the teaching of the subjects contained in the Syllabus should be distributed over a period of three years in such order as best suits individual training-schools in preparing the pupil nurse for examination at the end of that period. The advantage of a scheduled scheme lies in the assistance it offers to those training-schools which will be interchanging nurses under a scheme for reciprocal or affiliated training. It is obvious that without a schedule it would be impossible to guarantee that the three years' course had been covered by pupils going from one institution to another.

Gloucester Royal Infirmary, which has 140 beds, with an average occupation of 95, is about to appoint a sister-tutor, who will be on the resident staff of the infirmary, and whose duties will be to give lectures to the probationers of the Gloucester Royal Infirmary and of the Cheltenham General Hospital. The latter hospital has 118 beds, with an average occupation of 93. Different views are taken as to whether it is possible for a sister-tutor who shares her time between two or more institutions in different towns to be able to give the continuous attention to the pupils' work which is held to be necessary. But obviously it is impossible for every hospital to have such an official of its own, both on account of the cost and because at present there are not sufficient women having the special training and qualification. The combined arrangement between Gloucester and Cheltenham will, therefore, be watched with interest.

The revised regulations for admission to the Territorial Force Nursing Service which have recently been issued now provide for service at home or overseas as required. An important alteration has been made in the age at which candidates may be enrolled. Previously it was between twenty-three and fifty; under the revised regulations the ages are between twenty-four and thirty-five, and candidates, who must be of British parentage or naturalised British subjects, must possess a certificate of not less than three years' training in a civil general hospital of not less than 100 beds, or a Poor-law infirmary recognised by the Ministry of Health as a nurses' training-school. A matron will be required to retire at the age of sixty, and a sister or staff nurse at the age of fifty-five, or earlier should either give up their civil nursing appointments; but the two latter, subject to the age of retirement, will be eligible for readmission on again taking up nursing work. We are glad to note that the pay and allowances show a substantial increase, the rate of pay in the case of a matron being £115, rising to £185; sister £75, raising to £85; and staff nurses £60, rising to £65. The board and washing allowance, which is subject to variation, is at present 3s. 6d. a day at home or abroad, which will be reduced to 7d. a day when free messing is provided. A special uniform allowance is also granted. The rates and conditions for the grant of gratuities are under consideration, those paid during the Great War for satisfactory service were: matron £40, sister £30, and staff nurse £20, with an additional 10s. for each month or portion of a month of every year of completed service. But the revised regulations state that no guarantee can be given that these rates will be adopted in any future war.

We congratulate Miss Donaldson, matron of the London Temperance Hospital, on her appointment as matron of the Glasgow Royal Infirmary in succession to Miss Melrose, who is retiring after thirty-five years' service, fourteen of which have been spent as matron. Miss Donaldson is well known in London, for she was for eleven years matron of the Mount Vernon Hospital, from which she retired in 1916. During the war she was matron of a large unit in Serbia, and later in France. She possesses energy and ideas in addition to her undoubted professional qualifications, and has recently been instrumental in establishing post-graduate classes for nurses at the London Temperance Hospital. She should have a congenial field of work in Glasgow, where she will find a keen and progressive interest in hospital and nursing matters. The Royal Infirmary is a modern and well-equipped hospital of recent construction, the new buildings having been opened in July 1914 by the King and Queen.

The nursing staff of King's College Hospital, who organised a bazaar and fête on Wednesday and Thursday of last week, were evidently determined that everyone should have his money's worth of enjoyment. The Mathew Whiting and the Twining wards were transformed into gay scenes with bunting; and there were brightly decorated stalls offering flowers and fruit and pleasant country fare, children's clothes and jumpers, cakes and sweets, and all kinds of wares to tempt the visitor. The resident medical officers were kept busy on those two hot days serving ice-creams in the main corridor, while the roof-garden and the nurses' dining-hall

**ROUND THE HOSPITALS—(*continued*).**

were well filled with visitors taking tea. From five to nine dancing was allowed in the Sambrooke Ward, while out of doors there were donkeys, hidden treasure, bran-tubs, and many concerts and entertainments. The sale was opened on the first day by Dame Margaret Lloyd George, and on the second by the Mayoress of Camberwell.

We regret that the latest report of Miss Cox-Davies' condition remains grave, and her condition shows no change.

The Steyning Board of Guardians have decided unanimously in favour of the issue of a modified form of certificate, signed by the chairman, to the two nurses whose certificates, it will be remembered, the medical officer declined to sign. The certificate sets out that it was reported by the examiners to the Board of Guardians, at their meeting held on April 12, 1921, that . . . . successfully passed their examination as probationer nurses prior to the expiration of the period of their training. The Rev. T. Miller strongly protested against any suggestion that there was something against the nurses' characters. It would be interesting to know whether the above certificate would admit to registration.

The Dudley Dispensary is to be amalgamated with the Badley Memorial Home for District Nurses, and at the meeting held to consider the scheme at the Town Hall, Dudley, Mr. Whitehouse, the Hon. Secretary, presented a very interesting report. The Memorial Home was established by the Misses Badley in 1903, and is an illuminating example of the extent to which private benevolence can avail for the betterment of social conditions. These ladies have never made any appeal for funds, but have carried on the provision of district nurses for the sick poor entirely out of their own resources. The proposal now made is that the district nurses should work under the dispensary doctors, and that the work should be extended in various directions under united management. The Misses Badley have most generously entered into a covenant whereby they will set aside a fund to provide £500 in perpetuity towards the upkeep of the joint institution. The dispensary is to be adapted as a Home for the nurses, and an appeal for further support has been warmly received. It is hoped to adapt the dispensary and the Home to modern needs in regard to National Health Insurance.

The death is recorded of Miss Anna Acton Gwyn, a former matron at the York County Hospital. On the death of her mother, Miss Gwyn determined to devote her life to nursing the sick and suffering. She was trained at the Royal Hants Hospital, Winchester, and rapidly rose to the post of sister. She became lady superintendent at the Bath Hospital, Harrogate, and in 1894 was appointed matron at the York County Hospital, where she remained until 1901. She then returned to her training-school as matron, and subsequently became matron of the Nurses' Institute, Canterbury, from which post she retired after twelve years' service owing to ill-health. Miss Gwyn was a deeply religious woman, and exercised a marked influence on the members of her staffs by whom she will be long remembered.

Miss Lizzie Stewart, R.R.C., matron of the City of London Infirmary, whose retirement we recently announced, was presented with a handsome gift in the Council Chamber of the infirmary on July 1. A large company of nurses, numbering over a hundred, were present, and when the main presentation was over Nurse Greig presented, on behalf of the staff, a handsome crocodile dressing-case with silver fittings and an ebony case of cutlery. There were numerous private gifts also, and nurses from many distant regions trained by Miss Stewart have sent letters of appreciation.

